# Comparison of Interleukin-6 Plasma Concentration in Multisystem Inflammatory Syndrome in Children Associated With SARS-CoV-2 and Pediatric Sepsis

**DOI:** 10.3389/fped.2021.756083

**Published:** 2021-11-15

**Authors:** Franco Diaz, Raúl Bustos B, Felipe Yagnam, Todd J. Karsies, Pablo Vásquez-Hoyos, Juan-Camilo Jaramillo-Bustamante, Sebastián Gonzalez-Dambrauskas, Michelle Drago, Pablo Cruces

**Affiliations:** ^1^Unidad de Paciente Crítico Pediátrico, Hospital El Carmen de Maipú, Santiago, Chile; ^2^Escuela de Medicina, Universidad Finis Terrae, Santiago, Chile; ^3^Red Colaborativa Pediátrica de Latinoamérica (LARed Network), Montevideo, Uruguay; ^4^Unidad de Paciente Crítico Pediátrico, Hospital Guillermo Grant Benavente, Concepción, Chile; ^5^UCI Pediátrica, Clínica Sanatorio Alemán, Concepción, Chile; ^6^Unidad de Paciente Crítico Pediátrico, Hospital Exequiel González Cortés, Santiago, Chile; ^7^Pediatric Critical Care Medicine, Nationwide Children's Hospital, Columbus, OH, United States; ^8^Sociedad de Cirugía de Bogotá Hospital de San José, FUCS, Bogotá, Colombia; ^9^Universidad Nacional de Colombia, Bogotá, Colombia; ^10^Pediatric Intensive Care Unit, Hospital General de Medellín, Medellin, Colombia; ^11^Department of Pediatrics, University of Antioquia, Medellín, Colombia; ^12^Cuidados Intensivos Pediátricos Especializados (CIPe), Casa de Galicia, Montevideo, Uruguay; ^13^Facultad de Medicina, Unidad de Cuidados Intensivos de Niños del Centro Hospitalario Pereira Rossell (UCIN-CHPR), Universidad de la República, Montevideo, Uruguay; ^14^Centro de Investigación de Medicina Veterinaria, Escuela de Medicina Veterinaria, Facultad de Ciencias de la Vida, Universidad Andres Bello, Santiago, Chile

**Keywords:** COVID-19, MIS-C, sepsis, interleukin-6 (IL-6), shock

## Abstract

**Importance:** Multisystem Inflammatory Syndrome in Children (MIS-C) associated with SARS-CoV-2 infection is thought to be driven by a post-viral dysregulated immune response, where interleukin 6 (IL-6) might have a central role. In this setting, IL-6 inhibitors are prescribed as immunomodulation in cases refractory to standard therapy.

**Objective:** To compare plasma IL-6 concentration between critically ill children with MIS-C and sepsis.

**Design:** A retrospective cohort study from previously collected data.

**Setting:** Individual patient data were gathered from three different international datasets.

**Participants:** Critically ill children between 1 month-old and 18 years old, with an IL-6 level measured within 48 h of admission to intensive care. Septic patients were diagnosed according to Surviving Sepsis Campaign definition and MIS-C cases by CDC criteria. We excluded children with immunodeficiency or immunosuppressive therapy.

**Exposure:** None.

**Main Outcome(s) and Measure(s):** The primary outcome was IL-6 plasma concentration in MIS-C and sepsis group at admission to the intensive care unit. We described demographics, inflammatory biomarkers, and clinical outcomes for both groups. A subgroup analysis for shock in each group was done.

**Results:** We analyzed 66 patients with MIS-C and 44 patients with sepsis. MIS-C cases were older [96 (48, 144) vs. 20 (5, 132) months old, *p* < 0.01], but no differences in sex (41 vs. 43% female, *p* = 0.8) compared to septic group. Mechanical ventilation use was 48.5 vs. 93% (*p* < 0.001), vasoactive drug use 79 vs. 66% (*p* = 0.13), and mortality 4.6 vs. 34.1% (*p* < 0.01) in MIS-C group compared to sepsis. IL-6 was 156 (36, 579) ng/dl in MIS-C and 1,432 (122, 6,886) ng/dl in sepsis (*p* < 0.01), while no significant differences were observed in procalcitonin (PCT) and c-reactive protein (CRP). 52/66 (78.8%) patients had shock in MIS-C group, and 29/44 (65.9%) had septic shock in sepsis group. Septic shock had a significantly higher plasma IL-6 concentration than the three other sub-groups. Differences in IL-6, CRP, and PCT were not statistically different between MIS-C with and without shock.

**Conclusions and Relevance:** IL-6 plasma concentration was elevated in critically ill MIS-C patients but at levels much lower than those of sepsis. Furthermore, IL-6 levels don't discriminate between MIS-C cases with and without shock. These results lead us to question the role of IL-6 in the pathobiology of MIS-C, its diagnosis, clinical outcomes, and, more importantly, the off-label use of IL-6 inhibitors for these cases.

## Background

Multisystem Inflammatory Syndrome in Children (MIS-C) associated with SARS-CoV-2 infection is uncommon, but it is life-threatening, frequently presenting as rapid-onset severe organ failure (1, 2). It is thought to be driven by a post-viral dysregulated immune response, although the underlying pathophysiological processes are still poorly understood (2, 3). The clinical features of MIS-C have led to the assumption that the inflammation is exceptionally high, so-called cytokine storm; thus, empirical immunomodulation is the current therapy (2–6). However, there is no clear definition for cytokine storm. In theory, it refers to a harmful level of cytokines. However, as pointed out by some authors' the term is misleading because it falsely gives some impression to be related to a different entity, the cytokine release syndrome (CRS). CRS is a severe systemic inflammatory response caused by cytokines released by infused Chimeric antigen receptor (CAR)-modified T cells (CAR-Ts), where IL-6 is one of the main drivers (7).

Interleukin-6 (IL-6) is a pleiotropic cytokine with a pivotal role in the inflammatory and anti-inflammatory pathways. It has been previously studied in many conditions characterized by local or systemic insults, like bacterial and viral sepsis, and autoimmune diseases, like juvenile rheumatoid arthritis (JRA) (8).

Data regarding interleukin-6 (IL-6) in MIS-C is poor, but there is a growing interest in IL-6 inhibitors given the improved outcomes in COVID-19 adults treated with tocilizumab (6). However, off-label use of this drug in children without supporting evidence is of concern because of the side effects, particularly during an acute illness (9, 10).

With these thoughts in mind, we compared IL-6 plasma concentration in two conditions characterized by a dysregulated immune response: MIS-C and bacterial sepsis in children. We hypothesized that IL-6 concentrations would be higher in MIS-C than sepsis, especially if shock was present.

## Methods

### Design

Non-concurrent Cohort Study.

### Setting

Patient data were gathered from 3 different databases. The first group included 44 Pediatric Intensive Care Unit admissions due to sepsis before the COVID-19 pandemic (11). We collected 27 cases with MIS-C from Yagnam et al. (12) and 39 patients from the Critical Coronavirus and Kids Epidemiological Study (CAKE) (13) for the MIS-C group.

### Participants

Critically ill children between 1 month and 18 years of age, with an IL-6 level measured within 48 h of admission. Septic patients were diagnosed according to Surviving Sepsis Campaign definition (14). MIS-C cases were diagnosed by CDC criteria (15). We excluded children with immunodeficiency or immunosuppressive therapy.

### Variables

We recorded demographics, inflammatory biomarkers at admission, and outcomes. We analyzed the subgroup of shock, defined as any dose of epinephrine, norepinephrine, milrinone, or dobutamine plus dopamine >4 mcg/kg/min.

### Sample Size

We hypothesized that IL-6 levels are higher in MIS-C compared to sepsis. We estimated 37 cases per group, assuming a difference ≥ 100 ± 150 ng/mL, type I (α) error of 5%, and type II (β) error of 20%.

### Statistics

Continuous data were presented with median and interquartile range (IQR) and nominal data with frequencies. Wilcoxon signed-rank test and Pearson's chi (2) test were performed for comparisons. Significance was set at *p* < 0.05.

## Results

We analyzed 66 patients with MIS-C and 44 patients with sepsis. Patients with MIS-C were older and had lower severity scores compared to sepsis (Table 1). Pneumonia (34%), meningitis (21%), and bacteremia (18%) were the most frequent diagnosis in septic patients. MV was more frequent in the septic group, and there were no differences in vasoactive drug use. IL-6 was ≥16 pg/ml in 54/66 (82%) of MIS-C and 41/44 (93%) of septic patients (*p* = 0.154). CRP was ≥75mg/L in 25/63 (40%) of MIS-C and in 24/40 (60%) of septic group (*p* = 0.044). IL-6 was significantly higher in sepsis than MIS-C (*p* < 0.01), while CRP and PCT were non-statistically different (Table 1).

**Table 1:** Comparative analysis of demographics, laboratory, severity, and outcomes among cases with Multisystem Inflammatory Syndrome in Children (MIS-C) associated with SARS-CoV-2 and pediatric sepsis.

	**MIS-C**	***n* = 66**	**Sepsis**	***n* = 44**	***p***
**Demographics**					
Age (months)	96	(48–144)	**20**	**(5–132)**	<0.001
0–5 yo (*n*, %)	26	39.4%	**27**	**62.8%**	0.040
6–12 yo (*n*, %)	30	45.5%	10	23.3%	
13–19 yo (*n*, %)	10	15.2%	6	14.0%	
Female (*n*, %)	27	40.9%	19	43.2%	0.813
SARS-CoV-2 RT-CRP	51	77.3%			
SARS-CoV-2 Serology	44	66.6%			
**Laboratory**					
CRP (ng/ml)	30	(14–140)	94	(46–213)	0.088
Missing	3		4		
PCT (ng/dL)	4.7	(2.0–24.5)	3.1	(1.0–27.5)	0.830
Missing	10				
WBC (10^3^ cells/ml)	12.8	(7.5–18.1)	10.1	(4.7–20.7)	0.190
Missing	2		6		
Platelets (10^3^ cells/ml)	163	(118–231)	134	(49–302)	0.329
Missing	2		5		
IL-6 (pg/ml)	156	(36–579)	**1,432**	**(122–6,886)**	<0.001
**Severity Score**					
PELOD-2	**5**	**(2–11)**	12	(11–22)	<0.001
Missing	7		3		
**Outcomes**					
Mortality (*n*, %)	3	4.60%	**15**	**34.1%**	<0.001
Mechanical ventilation (*n*, %)	32	48.5%	**41**	**93.2%**	<0.001
Vasoactive drugs (*n*, %)	52	78.8%	29	65.9%	0.133

*Data are expressed in median (IQR) unless otherwise indicated*.

*yo, years old; RT-PCR, reverse transcriptase chain reaction; CRP, C-reactive protein; PCT, procalcitonin; WBC, white blood cell count; IL-6, interleukin 6; PELOD-2, Pediatric Logistic Organ Dysfunction score 2. Statistically significant differences between groups are shown in bold*.

**Figure 1:**
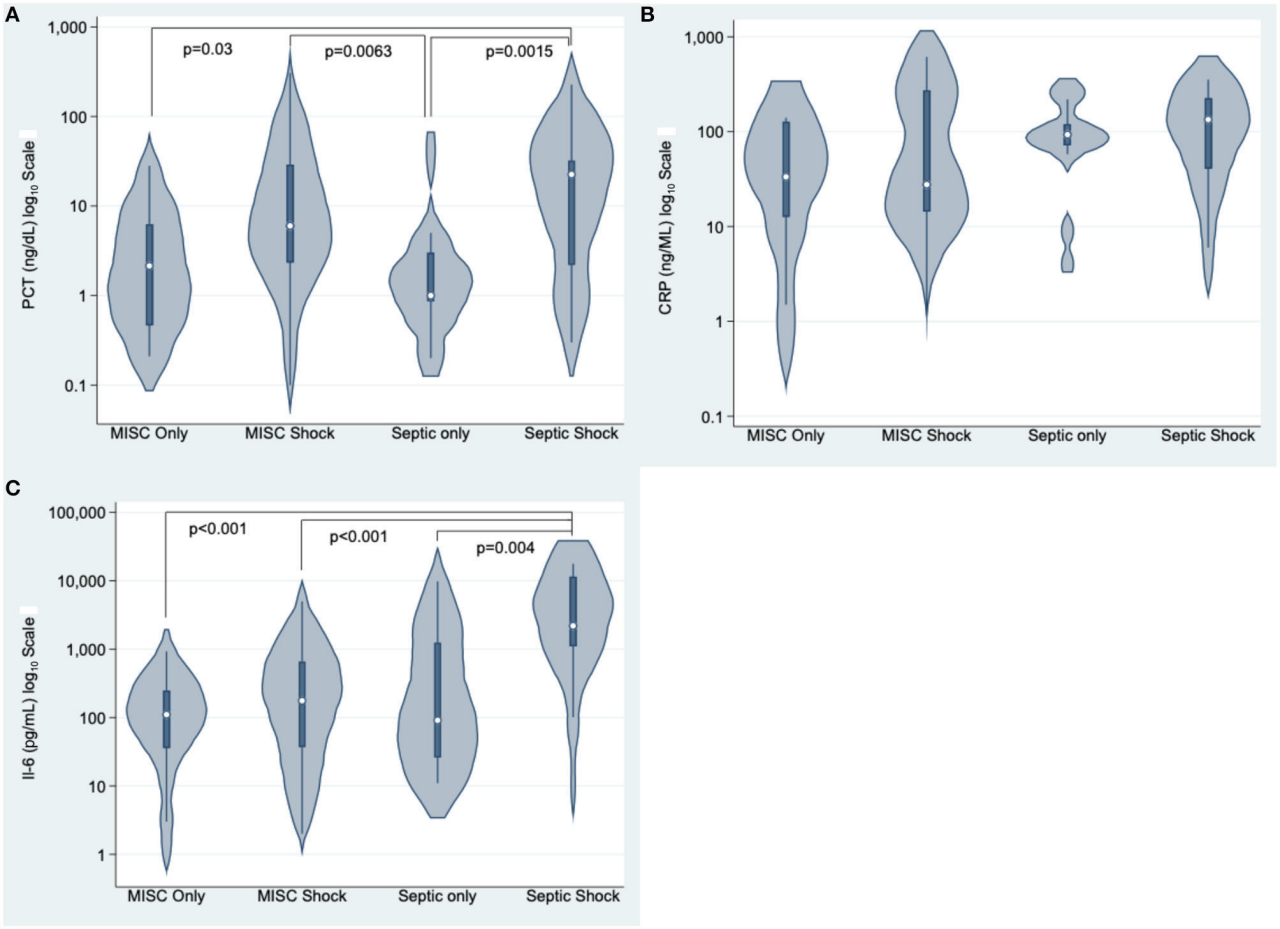
Procalcitonin (PCT, ng/dl) **(A)**, C-reactive protein (CRP, ng/ml) **(B)**, and Interleukin 6 (IL-6, pg/ml) **(C)** in critically ill children with MIS-C, MIS-C shock, sepsis, and septic shock. *p* values after Dunn's test with Bonferroni correction for group comparisons, *p* < 0.05 are significant.

52/66 (78.8%) patients had shock in MIS-C group, and 29/44 (65.9%) had septic shock in sepsis group. Septic shock group had a significantly higher plasma IL-6 concentration than the three other groups. Differences in IL-6, CRP, and PCT were not statistically different between MIS-C with and without shock (Figure 1).

Mortality was 34% in the sepsis group and 4.6% in the MIS-C group (*p* < 0.01).

## Discussion

IL-6 and other inflammatory markers were frequently elevated in critically ill children with MIS-C. However, in contrast to our hypothesis, we did not find differences in plasma IL-6 concentration between MIS-C with and without shock. Remarkably, IL-6 plasma concentrations in MIS-C were lower than in children with septic shock. Other inflammatory markers did not differ between MIS-C and sepsis. The role of IL-6 in MIS-C, and its potential target for therapy, is still obscure, probably given the heterogeneity of the disease and our poor understanding of the molecular pathophysiologic pathways involved.

The optimal management of MIS-C is still uncertain (4, 5, 15, 16). There are still incomplete data of the cohorts reported, and current definitions fail to assess the severity of cases, therapy effectiveness, and the risk of death and complications (5, 12, 16). Also, there are no randomized trials that have evaluated therapies for MIS-C. Several studies report high plasma cytokines in MIS-C, but its specific immune signature is still unknown (3). Our findings are in line with previous studies that described only moderately elevated IL-6 in MIS-C, even in severe cases (17). The IL-6 levels we and others have observed do not differentiate MIS-C from other inflammatory conditions, like macrophage activation syndrome, hemophagocytic lymphohistiocytosis, or Kawasaki disease with shock (18). Furthermore, other cytokines were found to differentiate MIS-C and KD with shock or pediatric COVID-19, like IL-10 and TNFα. However, unlike adult cases of COVID-19, IL-6 has not been directly associated with severity, and we were not able to demonstrate a higher IL-6 in MIS-C patients with shock. Abrams et al. (19) recently analyzed 1,080 MIS-C patients, reporting that many inflammatory markers, including CRP, ferritin, and IL-6 levels, were associated with PICU admission and shock. In that study, the median IL-6 plasma levels of MIS-C were within the same range as previous reports (between 100 to 200 pg/ml) (20–23).

It is still unknown if tocilizumab's beneficial effects are dependent on plasma IL-6 concentration. However, it is essential to recall that pediatric autoimmune diseases treated with tocilizumab, like systemic JRA, have robust translational and clinical research to support IL-6 as the main inflammatory promotor. In addition, IL-6 is a diagnostic and prognostic biomarker and a dynamic marker of the disease activity in this setting (24, 25). For instance, Vilaiyuk et al. described that median serum IL-6 levels were 82.2 (IQR 102.3) pg/ml in patients with systemic JRA and 19.9 (IQR 63.3) pg/ml in patients with arthritis.

Our finding that IL-6 and other inflammatory markers were not different in septic patients without shock and MIS-C is especially relevant when considering the differential diagnosis in a critically ill child and highlights the urgency to improve current case definitions. The inability to distinguish MIS-C from sepsis using IL-6, CRP, and PCT could delay immunomodulatory treatment for MIS-C or inappropriate immunosuppression in sepsis. In addition, as the COVID-19 pandemic subsides, an increasingly vaccinated population may result in many more seropositive children. Thus, the strategy of relying on serology and exposure to COVID-19 to trigger time-sensitive interventions for critically ill children may become problematic.

Our results also raise concern regards the widespread off-label use of IL-6 blockers in MIS-C. Although hyperinflammation is present in MIS-C, the term cytokine storm should be abandoned because it is misleading. For instance, many cases of MIS-C have low IL-6, and it is unknown if anti-IL-6 drugs are helpful in this setting. Still, in other acute conditions where tocilizumab is used, like the cytokine release syndrome associated with CAR-T therapy, IL-6 is usually ≥1,000 pg/ml (26). On the other hand, despite the high IL-6 levels in septic shock, commonly >1,000 pg/ml, immunomodulation has failed to improve outcomes (27–30). Even more, SSC guidelines (or other guidelines) do not mention tocilizumab or another selective cytokine pathway blockade. After many decades, our current understanding of sepsis therapy is that searching for a drug as a magic bullet does not fit the complex underlying pathophysiologic processes. In the same way, MIS-C therapy needs to be balanced with the increased risk of nosocomial and opportunistic infections in the critically ill due to immunosuppression induced by biologic agents. Thus, research is urgent to understand the myriad of biological pathways altered in MIS-C, define the clinical and immune profile phenotypes, and propose safe and effective therapies accordingly.

Our study has some limitations. First, the differences in demographics and severity between both groups can lead to differences in IL-6 concentrations. Second, there was a wide dispersion of IL-6 values and overlap between groups. Third, the small number of cases might lead to type II error in our subgroup analysis. Finally, IL-6 is not systematically measured in MIS-C, so a selection bias might be present. Nevertheless, the multicenter nature is one of the strengths of our data.

In summary, our data show that IL-6 plasma concentration was elevated in critically ill MIS-C patients but at levels much lower than those of sepsis. Furthermore, IL-6 levels do not discriminate between MIS-C cases with and without shock. These results lead us to question the role of IL-6 in the pathobiology of MIS-C, its diagnosis, severity classification, and clinical outcomes, and, more importantly, the off-label use of IL-6 inhibitors for these cases.

## Data Availability Statement

The datasets generated during and/or analyzed during the current study are available from the corresponding author on reasonable request.

## Author Contributions

FD, RB, and PC conceived of the presented idea. FD, PV-H, and J-CJ-B developed the theory and planned the analysis. SG-D and TK verified the analytical methods. RB, FY, MD, TK, PV-H, and SG-D screened cases to meet inclusion criteria and gathered clinical information. FD, PC, and TK supervised the findings of this work. All authors discussed the results and contributed to the final manuscript. All authors approved the final version of the manuscript.

## Conflict of Interest

The authors declare that the research was conducted in the absence of any commercial or financial relationships that could be construed as a potential conflict of interest.

## Publisher's Note

All claims expressed in this article are solely those of the authors and do not necessarily represent those of their affiliated organizations, or those of the publisher, the editors and the reviewers. Any product that may be evaluated in this article, or claim that may be made by its manufacturer, is not guaranteed or endorsed by the publisher.
